# Freiburg Neuropathology Case Conference: A 2-Year-Old Child with Progressive Headache, Nausea, and Personality Change

**DOI:** 10.1007/s00062-026-01622-y

**Published:** 2026-02-18

**Authors:** Emile Wogram, Ursula Feige, Roland Roelz, Marco Prinz, Horst Urbach, Daniel Erny, Christian A. Taschner

**Affiliations:** 1 Departments of Neuropathology, Medical Centre - University of Freiburg, University of Freiburg, Freiburg, Germany; 2 Faculty of Medicine, University of Freiburg, Freiburg, Germany; 3 Department of Neuroradiology, Medical Centre - University of Freiburg, University of Freiburg, Freiburg, Germany; 4 Department of Neurosurgery, Medical Centre - University of Freiburg, University of Freiburg, Freiburg, Germany

## Case Report

A 2-year-old child presented with progressive headache, nausea, and personality changes which triggered cranial magnetic resonance maging (MRI) and revealed a large right parietal mass lesion. Given the size and mass effect, urgent surgical resection was indicated.

The patient was positioned supine with the head turned to the left. A parietal craniotomy including the central region was performed, and the dura was opened toward the sinus.

A superficially well-demarcated mass was encountered, markedly compressing the underlying brain. Frozen section analysis was consistent with a malignant small-cell tumour, likely a primitive neuroectodermal tumour. Tumour boundaries were progressively defined. While the lesion was well separated from adjacent cortex superficially, deeper infiltration into parenchyma was evident. The resection proceeded in an extralesional, glioma-like fashion. Two compartments were identified: a soft, suctionable main tumour mass, and a firm component tightly adherent to the sinus. After delineating the dorsal circumference and falx level, resection proceeded anteriorly under cortical and subcortical stimulation. Subcortical stimulation up to 20 mA elicited no motor responses, permitting safe tumour removal with safety margins posteriorly to the corticospinal tract.

The parenchymal component was completely resected; however, the firm portion adherent to the sinus could not be fully separated. Careful dissection allowed gradual thinning of this component down to the sinus wall, but complete (R0) resection was not feasible because of infiltration into the sinus wall. The infiltrated segment was coagulated as safely as possible and reinforced with a Duragen patch. Dural closure was achieved, the bone flap reattached, and layered wound closure completed. The postoperative course was uneventful, and on postoperative day 6 the child was discharged home in good general condition.

Postoperative staging revealed no evidence of spinal metastases, and cerebrospinal fluid (CSF) analysis was unremarkable. Systemic therapy according to the International Society of Paediatric Oncology–HIT (I-HIT) protocol, consisting of carboplatin and etoposide in combination with weekly intrathecal topotecan, was initiated. Magnetic resonance imaging re-evaluation after two cycles showed no evidence of tumour progression. Based on these findings, the decision was made to proceed with proton beam radiotherapy. One month after surgery the child no longer exhibited signs of increased intracranial pressure compared with his initial presentation. According to parental observation, behavioural changes had largely regressed, although increased aggression persisted with gradual improvement over time; no new neurological symptoms occurred during therapy, and physical endurance improved markedly without deficits noted during regular physiotherapy.

## Imaging

**Fig. 1:**
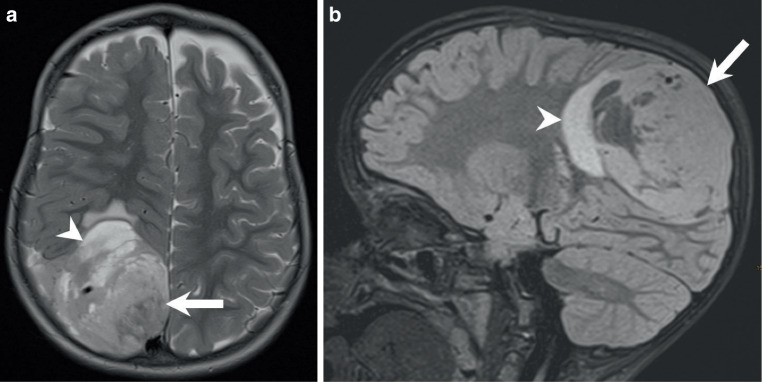
Axial T2-weighted MR images (**a**) showed a large right parietal lesion with a solid component (arrow) adjacent to cystic portions (arrowhead). On sagittal fluid-attenuated inversion recovery (FLAIR) images (**b**) the lesion appears isointense compared to the cerebral cortex (arrow). Surrounding perifocal edema is evident (arrowhead)

**Fig. 2:**
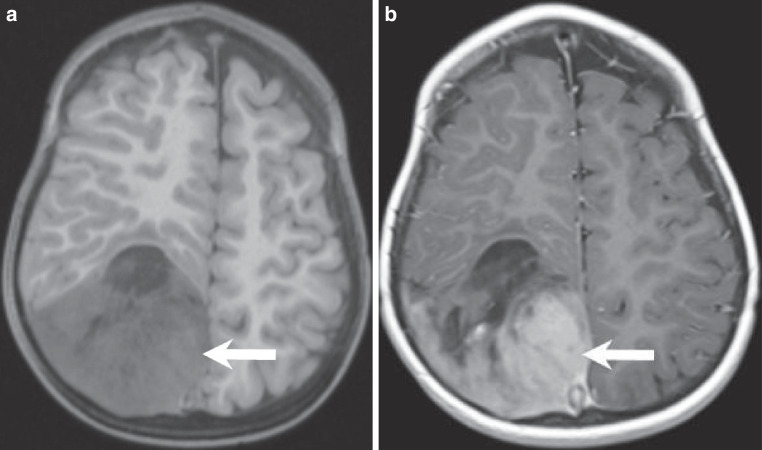
Axial pre-contrast T1-weighted MR images (**a**) revealed a hypointense signal of the solid tumour components (arrow). Axial T1 weighted images after gadolinium administration (**b**), showed marked contrast enhancement of the solid tumour portion

The MRI on admission revealed an extensive space-occupying intraaxial lesion in the right parietal lobe (Figs. 1 and 2). The mass showed both solid components (Figs. 1 and 2; arrows) and cystic portions (Fig. 1a, arrowhead), accompanied by pronounced perifocal oedema (Fig. 1b, arrowhead). Diffusion-weighted imaging demonstrated marked diffusion restriction, suggestive of hypercellularity (not shown). On T1-weighted images, the solid components appeared hypointense on native scans (Fig. 2a, arrow), with strong gadolinium enhancement after contrast administration (Fig. 2b, arrow). No evidence of calcification or haemorrhage was detected.

## Differential Diagnosis

Supratentorial, cortically based tumours with well-defined margins and diffusion restriction are rare in children [1–3]. In patients older than 1.5 years, this radiologic appearance is most consistent with either an embryonal tumour or an ependymoma [4]. High-grade gliomas are less likely because they typically demonstrate ill-defined margins: diffuse hemispheric glioma, H3G34-mutant, usually affects teenagers and young adults; paediatric diffuse high-grade glioma, H3 wild-type, IDH wild-type generally presents as a large supratentorial mass with poorly defined borders, often accompanied by haemorrhage and necrosis; and infant-type hemispheric glioma typically arises within the first year of life [4]. Desmoplastic infantile glioma (DIG) and desmoplastic infantile astrocytoma (DIA) are low-grade tumours occurring in infants under 2 years, characterized by large cortically based cystic-solid masses with heterogeneous enhancement, calcifications, and variable diffusivity (4 + 5). These entities are unlikely in the present case given the patient’s age.

### Supratentorial Ependymal Tumour

ZFTA (zinc finger translocation associated, previously known as C11orf95) fusion–positive ependymomas typically occur in children with a mean age of 7 years, whereas YAP1 fusion–positive tumours are less common and usually present within the first year of life. They manifest as large, well-delineated masses with thick, heterogeneous solid enhancement, prominent cystic components, central necrosis surrounded by calcifications and hemorrhage (the “periwinkle sign”), and associated peritumoural edema [4].

### Atypical Teratoid/Rhabdoid Tumour (ATRT)

ATRT is a rare, highly malignant embryonal tumour (WHO grade 4) that predominantly affects children younger than 2 years. Neuroimaging typically reveals cystic components, thick and irregular enhancement of the cyst wall, marked surrounding edema, and a strong tendency for dissemination at diagnosis. Calcifications and diffusely low ADC values are also frequent findings [4, 5].

### Embryonal Tumour with Multilayered Rosettes (ETMR)

ETMR is an aggressive WHO grade 4 embryonal tumour that primarily arises in infants under 3 years of age, with no clear sex predilection [6, 7] or a slight female predominance (59%, [2]). Approximately 70% occur in supratentorial locations, although other sites have been reported [2, 7]. These tumours are usually large and well demarcated. Notably, ETMRs often exhibit little to no enhancement and relatively limited peritumoural edema compared with their size [4]. On MRI, they demonstrate heterogeneous signal intensity with frequent diffusion restriction, cystic areas, and intratumoural hemorrhage [8]. Imaging features overlap with other CNS embryonal tumours, but ETMRs tend to have very large volumes (mean 115 cm^3^), often spanning multiple lobes [9]. Clinically, they behave aggressively with rapid progression despite intensive therapy. Local recurrence is most common, although leptomeningeal dissemination is frequent and rare extracranial relapses have been reported [10, 11].

### CNS Neuroblastoma, FOXR2-Activated

This large embryonal tumour typically presents in the first decade of life (mean age 5 years) and demonstrates cystic and/or necrotic components, heterogeneous enhancement, calcifications, and low mean ADC values [4]. With early and adequate treatment, prognosis can be relatively favorable [3].

### CNS Tumours with BCOR Internal Tandem Duplication

These WHO grade 4 tumours typically arise in early childhood (median age 3.5 years). Imaging shows large cortical masses with reduced diffusivity, variable signal heterogeneity, and poor contrast enhancement, often with central necrosis, hemorrhage, and calcifications. Prominent central veins within the tumour are a characteristic feature [12]. Like ETMR, these tumours occur in very young children and share overlapping imaging features, including the relative absence of peripheral edema [4].

## Histology, Immunohistochemistry and Molecular Pathology

**Fig. 3:**
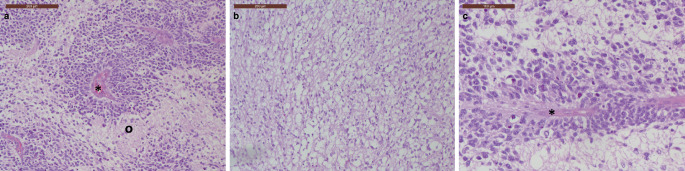
Haematoxylin-eosin-stained (H&E) section depicting multilayered rosettes, which is a defining feature of Embryonal tumour with multilayered rosettes (ETMR). The lumen of the rosette is indicated by an asterisk, the black circle indicates the surrounding neuropil (**a**). Another H&E stained section with abundant tumour surrounding neuropil (**b**). Scale bars: 200 µm. Higher magnification of a H&E stained section featuring a multilayered rosette (**c**). The lumen of the rosette is indicated by an asterisk. Scale bar: 100 µm

In haematoxylin–eosin (H&E)-stained sections of formalin-fixed, paraffin-embedded biopsy material, a tumour with increased cellularity was evident. It predominantly showed neuropil-rich areas interspersed with dense tumour-cell aggregates forming multilayered rosettes (Fig. 3a, b). These rosettes consisted of embryonal cells arranged as pseudostratified neuroepithelium surrounding a central lumen. Cells adjacent to the lumen displayed a defined apical surface, with nuclei located basally, away from the lumen (Fig. 3c). Focal regions of fresh haemorrhage were also present.

**Fig. 4:**
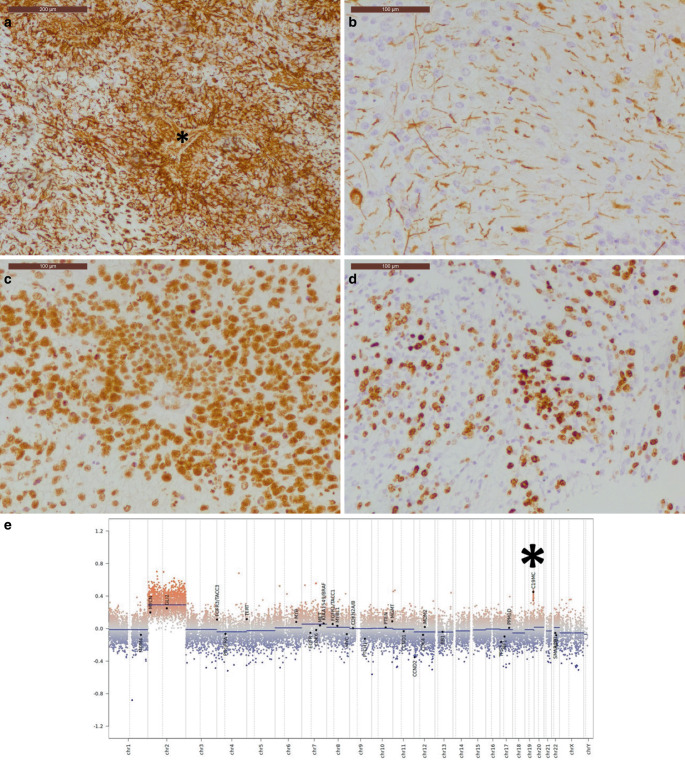
The embryonal component of the tumour shows a strong positivity in the immunohistochemistry against vimentin. The lumen of a multilayered rosette is indicated by an asterisk (**a**). Scale bar: 200 µm. The neuropil surrounding the embryonal component of the tumour shows a glial cell elements that strongly react in the immunohistochemistry against GFAP (**b**). The embryonal component of the tumour exhibits a strong positivity in the immunohistochemistry against Ini-1 (**c**). The proliferation marker MIB‑1 indicates a high proliferation rate of the tumour (**d**). Scale bars **d,** **e**: 100 µm. DNA methylation profile (**e**) with alterations of a microRNA cluster on chromosome 19q13.42 (C19M, indicated by an asterisk)

Immunohistochemistry demonstrated rosettes and tubular structures strongly positive for vimentin (Fig. 4a). Glial cell elements within the neuropil stained positively for GFAP (Fig. 4b). Nuclear expression of INI1 was retained (Fig. 4c). The proliferation marker MIB‑1 revealed a high proliferative index of up to 80% (Fig. 4d).

DNA methylation profiling classified the tumour as ETMR, showing gains on chromosome 2 and focal amplification of chromosome 19q (Fig. 4e). The calibrated score was 0.99983 for the methylation class “embryonal tumour with multilayered rosettes, C19MC-altered”, according to the Brain Tumour Classifier (version 12.8), where a score ≥ 0.9 indicates a robust match.

### Diagnosis

- Embryonal tumour with multilayered rosettes (ETMR), CNS WHO Grade 4

ETMR, formally also known as ETANTR (Embryonal Tumour with Abundant Neuropil and True Rosettes) is an aggressive WHO grade 4 embryonal tumour that primarily arises in infants under 3 years of age, with no clear sex predilection [6, 7] or a slight female predominance (59%, 2) Three morphological patterns are recognized: embryonal tumour with abundant neuropil and true rosettes, ependymoblastoma, and medulloepithelioma [13]. The embryonal components, including rosettes and tubular structures, typically show strong immunoreactivity for nestin and vimentin [13–15]. Approximately 90% of ETMRs harbour C19MC alterations at chromosome 19q13.42 [6, 16], while ~5% are associated with DICER1 mutations [17]. Diagnostic confirmation requires identification of an embryonal CNS tumour with one of the characteristic ETMR histological patterns, together with either C19MC alteration, DICER1 mutation, or, in unresolved cases, a DNA methylation profile consistent with ETMR.

Although most ETMRs are intracranial, with 45% arising in non-hemispheric sites, spinal cord cases are rare [13]. Reliable epidemiological data are lacking, but CNS embryonal tumours overall—previously grouped under the designation CNS-PNET—are diagnosed in roughly 1 per 700,000 children aged 0–4 years [7, 18].

Important differential diagnoses include other embryonal tumours such as atypical teratoid/rhabdoid tumour (ATRT) and medulloblastoma, which differ in histology and immunophenotype, as well as rare entities like intraocular medulloepithelioma and sacrococcygeal ependymoblastoma. These latter tumours share some histopathological features with ETMR but exhibit distinct molecular alterations and are therefore considered separate entities [13].

The prognosis of ETMR is poor due to its rapid growth. Median survival is approximately 12 months despite multimodal therapy. Aggressive management with gross total resection, radiotherapy, and high-dose chemotherapy may prolong survival in selected cases [13, 19].

## Data Availability

No datasets were generated or analysed during the current study.

## References

[1] McNamara C, Mankad K, Thust S, Dixon L, Limback-Stanic C, D’Arco F, et al. 2021 WHO classification of tumours of the central nervous system: a review for the neuroradiologist. Neuroradiology. 2022;64(10):1919–50. 35869291 10.1007/s00234-022-03008-6

[2] Khan S, Solano-Paez P, Suwal T, Lu M, Al-Karmi S, Ho B, et al. Clinical phenotypes and prognostic features of embryonal tumours with multi-layered rosettes: a rare brain tumour registry study. Lancet Child Adolesc Health. 2021;5(11):800–13. 34599879 10.1016/S2352-4642(21)00245-5

[3] Schepke E, Löfgren M, Pietsch T, Kling T, Nordborg C, Olsson Bontell T, et al. Supratentorial CNS-PNETs in children; a Swedish population-based study with molecular re-evaluation and long-term follow-up. Clin Epigenetics. 2023;15:40. 36895035 10.1186/s13148-023-01456-2 PMC9996973

[4] Rameh V, Löbel U, D’Arco F, Bhatia A, Mankad K, Poussaint TY, et al. Cortically based brain tumours in children: A decision-tree approach in the radiology reading room. AJNR Am J Neuroradiol. 2025;46:11–23. 39181692 10.3174/ajnr.A8477 PMC11735440

[5] Koelsche C, Sahm F, Paulus W, Mittelbronn M, Giangaspero F, Antonelli M, et al. BRAF V600E expression and distribution in desmoplastic infantile astrocytoma/ganglioglioma. Neuropathol Appl Neurobiol. 2014;40(3):337–44. 23822828 10.1111/nan.12072

[6] Lambo S, Gröbner SN, Rausch T, Waszak SM, Schmidt C, Gorthi A, et al. The molecular landscape of ETMR at diagnosis and relapse. Nature. 2019;576:274–80. 31802000 10.1038/s41586-019-1815-x PMC6908757

[7] Lambo S, von Hoff K, Korshunov A, Pfister SM, Kool M. ETMR: a tumour entity in its infancy. Acta Neuropathol. 2020;140:249–66. 32601913 10.1007/s00401-020-02182-2 PMC7423804

[8] Nowak J, Seidel C, Berg F, Pietsch T, Friedrich C, von Hoff K, et al. MRI characteristics of ependymoblastoma: results from 22 centrally reviewed cases. AJNR Am J Neuroradiol. 2014;35(10):1996–2001. 24948504 10.3174/ajnr.A4002 PMC7966246

[9] Nowak J, Seidel C, Pietsch T, Alkonyi B, Fuss TL, Friedrich C, et al. Systematic comparison of MRI findings in pediatric ependymoblastoma with ependymoma and CNS primitive neuroectodermal tumour NOS. Neuro Oncol. 2015;17(8):1157–65. 25916887 10.1093/neuonc/nov063 PMC4490876

[10] Korshunov A, Sturm D, Ryzhova M, Hovestadt V, Gessi M, Jones DTW, et al. Embryonal tumour with abundant neuropil and true rosettes (ETANTR), ependymoblastoma, and medulloepithelioma share molecular similarity and comprise a single entity. Acta Neuropathol. 2014;128(2):279–89. 24337497 10.1007/s00401-013-1228-0 PMC4102829

[11] Shah AH, Khatib Z, Niazi T. Extracranial extra-CNS spread of embryonal tumour with multilayered rosettes (ETMR): case series and systematic review. Childs Nerv Syst. 2018;34(4):649–54. 29177676 10.1007/s00381-017-3657-x

[12] Cardoen L, Tauziède-Espariat A, Dangouloff-Ros V, Moalla S, Nicolas N, Roux CJ, et al. Imaging features with histopathologic correlation of CNS high-grade neuroepithelial tumours with a BCOR internal tandem duplication. AJNR Am J Neuroradiol. 2022;43(1):151–6. 34887247 10.3174/ajnr.A7367 PMC8757552

[13] WHO Classification of Tumours Editorial Board. Central nervous system tumours. 5 ed. Lyon: International Agency for Research on Cancer; 2021.

[14] Eberhart CG, Brat DJ, Cohen KJ, Burger PC. Pediatric neuroblastic brain tumours containing abundant neuropil and true rosettes. Pediatr Dev Pathol. 2000;3(4):346–52. 10890250 10.1007/s100249910049

[15] Gessi M, Giangaspero F, Lauriola L, Gardiman M, Scheithauer BW, Halliday W, et al. Embryonal tumours with abundant neuropil and true rosettes: a distinctive CNS PNET. Am J Surg Pathol. 2009;33(2):211–7. 18987548 10.1097/PAS.0b013e318186235b PMC4512670

[16] Kleinman CL, Gerges N, Papillon-Cavanagh S, Sin-Chan P, Pramatarova A, Quang DA, et al. Fusion of TTYH1 with the C19MC microRNA cluster drives DNMT3B isoform expression in ETMR. Nat Genet. 2014;46(1):39–44. 24316981 10.1038/ng.2849

[17] de Kock L, Priest JR, Foulkes WD, Alexandrescu S. An update on the CNS manifestations of DICER1 syndrome. Acta Neuropathol. 2020;139(4):689–701. 30953130 10.1007/s00401-019-01997-y

[18] Ostrom QT, Cioffi G, Gittleman H, Patil N, Waite K, Kruchko C, et al. CBTRUS Statistical Report: Primary Brain and Other CNS Tumours Diagnosed in the US in 2012–2016. Neuro Oncol. 2019;21(Suppl 5):v1–100. 31675094 10.1093/neuonc/noz150 PMC6823730

[19] Jaramillo S, Grosshans DR, Philip N, Varan A, Akyuz C, McAleer MF, et al. Radiation for ETMR: Literature review and case series of patients treated with proton therapy. Clin Transl Radiat Oncol. 2019;15:31–7. 30582019 10.1016/j.ctro.2018.11.002 PMC6297264

